# Lysosomes as chemical and metabolic organizers of central nervous system injury and repair

**DOI:** 10.3389/fchem.2026.1891677

**Published:** 2026-09-23

**Authors:** Tengfei Zhao, Hanxu Huang, Yifan Wang, Yuliang Zhong, Zhiwei Zhong, Deting Xue, Wei Zhu, Kan Xu

**Affiliations:** 1 Department of Orthopedic Surgery, The Second Affiliated Hospital, Zhejiang University School of Medicine, Hangzhou, Zhejiang Province, China; 2 Orthopedics Research Institute of Zhejiang University, Hangzhou, Zhejiang Province, China; 3 Key Laboratory of Motor System Disease Research and Precision Therapy of Zhejiang Province, Hangzhou, Zhejiang Province, China; 4 Clinical Research Center of Motor System Disease of Zhejiang Province, Hangzhou, Zhejiang Province, China; 5 Department of Cardiology, The Second Affiliated Hospital, School of Medicine, Zhejiang University, Hangzhou, China; 6 State Key Laboratory of Transvascular Implantation Devices, Hangzhou, China; 7 Heart Regeneration and Repair Key Laboratory of Zhejiang Province, Hangzhou, China

**Keywords:** central nervous system, lysosome, metabolism, neural repair, neuroinflammation

## Abstract

Lysosomes are degradative, metabolic and signaling organelles in central nervous system (CNS) cells and play essential roles in neural homeostasis, injury responses and disease progression. Changes in lysosomal function are required for neurons, microglia, astrocytes and oligodendrocyte-lineage cells to adapt to proteotoxic stress, lipid overload, mitochondrial damage and inflammatory stimulation. Therefore, lysosomal activity in the CNS is regulated by diverse chemical and cellular cues, including luminal acidification, hydrolase maturation, ion transport, membrane integrity, lipid handling and transcriptional lysosome biogenesis. Here, we summarize current knowledge of lysosomal organization and function in neural cells, including lysosomal membrane signaling, vacuolar H^+^-ATPase (V-ATPase)-dependent acidification, ion and redox regulation, lysosomal membrane quality control, and metabolic recycling of proteins, lipids, glycans and damaged organelles. We then survey pathological lysosomal remodeling in spinal cord injury, traumatic brain injury, Alzheimer’s disease, Parkinson’s disease and related neurological disorders. Across these conditions, lysosomal remodeling may initially support cargo clearance and stress adaptation, but becomes maladaptive when degradative capacity, membrane repair or lipid-processing ability fails to meet cellular demand. Finally, we discuss lysosome-oriented therapeutic strategies for neural repair, including restoration of lysosomal acidification and hydrolase activity, transcription factor EB/transcription factor E3 (TFEB/TFE3)-mediated lysosomal biogenesis, regulation of lysosomal ion channels, enhancement of myelin lipid turnover, and lysosome-responsive nanomedicine. This Review highlights lysosomes as active organizers of CNS injury and repair rather than passive endpoints of degradation.

## 1 Introduction

Lysosomes were once regarded mainly as terminal degradative compartments, but this view is no longer sufficient for the central nervous system (CNS). In addition to degrading macromolecular cargo, lysosomes contribute to nutrient sensing, ion homeostasis, membrane trafficking, and organelle quality control (Nixon and Rubinsztein, 2024; Xun and Tan, 2025). This broader view is especially relevant to neural tissue. Long-lived neurons, microglia, and astrocytes all depend on lysosomes, which help these cells maintain proteostasis, recycle damaged organelles, and process injury-associated cargo.

Conceptually, the conventional degradative model places lysosomes at the downstream end of endocytic, phagocytic, and autophagic pathways: cargo enters an acidic, hydrolase-rich lumen, is broken down, and its constituents are returned to the cytosol. Although fundamental, this model primarily describes cargo clearance and recycling as outputs of lysosomal activity. The chemical and metabolic organizer framework instead considers how the biochemical state of lysosomes couples cargo processing to metabolic and signaling responses. Luminal pH, hydrolase maturation, ion and redox balance, membrane integrity, and lipid-processing capacity determine not only whether cargo is cleared, but also which metabolites are recovered and which nutrient-sensing, stress-response, and inflammatory pathways are engaged. Lysosomes can therefore influence cell state before overt degradative failure, and increased lysosomal biogenesis or marker abundance may indicate either successful adaptation or unresolved stress. In CNS injury and disease, this framework links organelle chemistry to cell-type-specific outcomes, including neuronal proteostasis, microglial debris clearance and the resolution of inflammation, astrocytic matrix remodeling, and oligodendrocyte-lineage lipid turnover and remyelination (Ballabio and Bonifacino, 2020; Lawrence and Zoncu, 2019; Udayar et al., 2022).

In the CNS, lysosomal function cannot be inferred solely from an increase in the number of lysosome-associated membrane protein 1 (LAMP1)-positive lysosomes or the accumulation of microtubule-associated protein 1 light chain 3 (LC3)-positive autophagosomes. Lysosomes may be enlarged yet insufficiently acidified, hydrolase-rich yet fusion-incompetent, or transcriptionally expanded yet unable to complete degradation. Therefore, lysosomal remodeling should be understood as a functional state rather than a static marker phenotype. This distinction is important after spinal cord injury and traumatic brain injury. In these cases, cells face damaged mitochondria, axonal fragments, extracellular matrix debris, myelin lipids and redox-active substrates. This cargo may be degraded, recycled, or converted into danger signals. This outcome depends on several lysosomal functions, including acidification, hydrolase maturation, membrane integrity, ion transport, and lipid-processing capacity (Cen et al., 2024; Qin et al., 2023).

This functional perspective has also changed our understanding of glial responses. Microglia and infiltrating macrophages are often described as inflammatory effector cells, but their repair potential depends to a large extent on lysosome-dependent phagolysosomal processing ability. In experimental spinal cord injury, microglia support spontaneous recovery and coordinate responses among resident glial cells and infiltrating leukocytes (Brennan et al., 2022). Therefore, the key question is not whether microglia are activated, but whether phagocytic uptake can be matched with efficient lysosomal digestion, lipid metabolism and inflammation subsidence. When this coupling fails, myelin-rich debris and redox substrates may overload lysosomes and maintain lipid storage, oxidative damage and chronic neuroinflammation.

At the molecular level, lysosomes are not only degradative organelles, but also signaling platforms. Their surface carries the nutrient-sensing system, especially the mechanistic target of rapamycin complex 1 (mTORC1)–transcription factor EB/transcription factor E3 (TFEB/TFE3) axis. This axis links the availability of amino acids with autophagy and lysosomal biogenesis (Cui et al., 2023a). Under the conditions of starvation, lysosomal stress or degradation defects, TFEB/TFE3 activation can promote lysosomal renewal and autophagic adaptation. However, this reaction is context-dependent: in some cases, lysosomal biogenesis may restore degradation capacity; in other cases, it may also reflect unresolved lysosomal stress. Therefore, increased lysosome-related transcription should not be equated with functional recovery unless acidification, hydrolase activity, cargo degradation and membrane integrity are also restored.

Lysosomal chemistry further determines whether CNS cells adapt to stress or progress toward degeneration. Luminal acidification enables hydrolase maturation and substrate degradation; lysosomal Ca^2+^ and K^+^ fluxes regulate trafficking, fusion and transcriptional adaptation; and redox-active metals such as iron may either be recycled safely or promote lipid peroxidation when lysosomal buffering fails (Cen et al., 2024; Huang et al., 2025). In CNS injury, hemorrhage, demyelination, mitochondrial damage and inflammatory oxidants increase the burden of redox-active and lipid-rich substrates. In this setting, lysosomes are active regulators of oxidative injury, inflammatory amplification and cell fate, rather than passive storage compartments.

These concepts have direct therapeutic implications. Lysosome-oriented therapy should not be reduced to broad autophagy activation, because increasing autophagosome formation may worsen pathology when downstream acidification, fusion or degradation is defective. More precise strategies should target the rate-limiting lysosomal module, including vacuolar H^+^-ATPase (V-ATPase)-dependent acidification, cathepsin maturation, lysosomal membrane repair, TFEB/TFE3-driven biogenesis, myelin lipid degradation, cholesterol efflux or lysosome-targeted delivery systems (Du et al., 2025; Lo et al., 2024; Zeng et al., 2023). The therapeutic goal is therefore not to increase lysosome number indiscriminately, but to restore degradative competence in the appropriate cell type and disease stage.

In this Review, we synthesize recent advances in lysosome biology from the perspective of CNS injury and neural repair. We first discuss lysosomal organization, including membrane architecture, luminal acidification, ion and redox regulation, and membrane quality control. We then examine lysosomes as metabolic recycling hubs in CNS cells, focusing on protein degradation, lipid turnover, glycan and extracellular matrix processing, and lysosome–mitochondria crosstalk. Next, we analyze pathological lysosomal remodeling in spinal cord injury, traumatic brain injury, Alzheimer’s disease, Parkinson’s disease and related neurological disorders. Finally, we evaluate lysosome-oriented therapeutic strategies, including restoration of acidification and hydrolase activity, TFEB/TFE3 modulation, ion-channel targeting, lipid-turnover reprogramming and lysosome-responsive nanomedicine. By integrating organelle chemistry, cell-type specificity and disease-stage dependence, this Review defines lysosomes as active organizers of neural repair rather than passive endpoints of degradation.

## 2 Molecular and chemical organization of lysosomes

Lysosomes are not merely terminal digestive vesicles. Instead, they are chemically compartmentalized organelles that can integrate macromolecular degradation with nutrient sensing, membrane trafficking, ion signaling, stress adaptation and cell fate control (Nowosad and Besson, 2022; Riederer et al., 2023; Savini et al., 2019). Lysosomal function depends on coordinated structural and biochemical organization, including highly glycosylated limiting membranes, acidic luminal environments rich in hydrolases, ion and metal transport systems, and membrane quality control pathways that can repair, reshape or remove damaged lysosomes (Xun and Tan, 2025; Yang and Tan, 2023). Recent studies have therefore redefined lysosomes as active metabolic, signaling, and quality-control organelles rather than passive degradative compartments. As summarized in Figure 1, lysosomal function is coordinated through four closely interconnected modules: membrane signaling, luminal acidification, ionic and redox regulation, and membrane quality control. Together, these modules determine cargo degradation, metabolic recycling, and cellular adaptation to stress, while their relative functional importance varies among CNS cell populations (Figure 1; Zhu et al., 2020).

**FIGURE 1:**
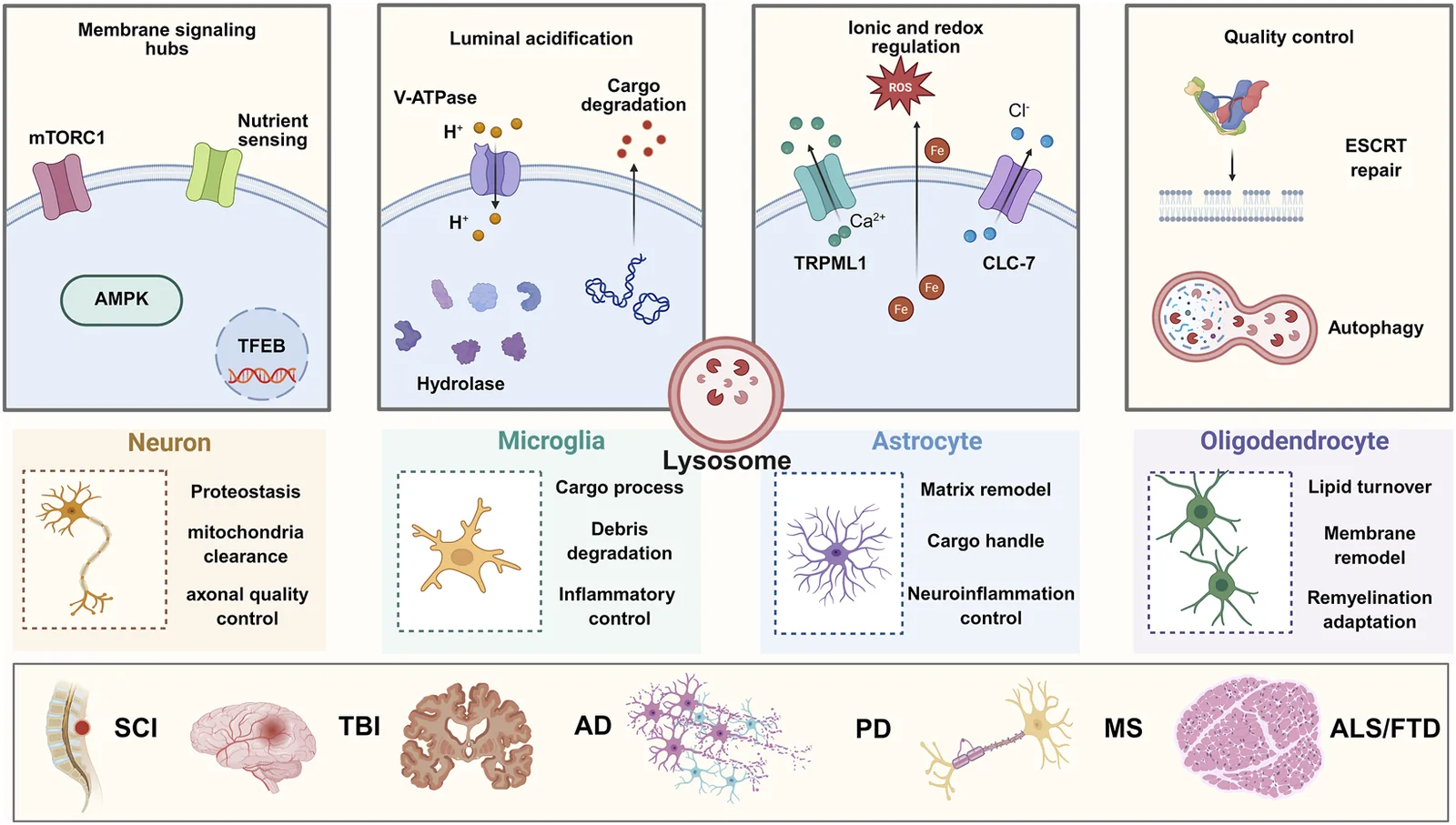
Lysosomal organization and cell-type-specific functions in central nervous system. Lysosomes act as chemical, metabolic and signaling organizers in central nervous system (CNS) injury and repair. This figure summarizes four key lysosomal modules: membrane signaling, luminal acidification, ion/redox regulation and membrane quality control. These modules participate in major CNS disorders, including spinal cord injury (SCI), traumatic brain injury (TBI), Alzheimer’s disease (AD), Parkinson’s disease (PD), multiple sclerosis (MS) and amyotrophic lateral sclerosis/frontotemporal dementia (ALS/FTD). In different CNS cell types, lysosomes regulate neuronal proteostasis and mitochondrial clearance, microglial debris degradation and inflammation, astrocytic matrix remodeling and inflammatory responses, and oligodendrocyte-lineage lipid turnover and remyelination. Created with BioRender.com, License Number: MC29RG9NAX.

### 2.1 Lysosomal membrane and signaling hubs

The lysosomal membrane functions simultaneously as a protective barrier and a signaling platform (Ballabio and Bonifacino, 2020). On the luminal side, highly glycosylated membrane proteins, including LAMP1 and lysosome-associated membrane protein 2 (LAMP2), form a glycocalyx-like protective layer that shields the limiting membrane from acidic hydrolases (Saftig and Klumperman, 2009). On the cytosolic side, the same membrane serves as a docking surface for trafficking regulators, nutrient-sensing machinery, small guanosine triphosphatases (GTPases), kinases and transcriptional-control complexes. This dual organization enables lysosomes to preserve a degradative lumen while relaying highly specific information to the cytoplasm (Lawrence and Zoncu, 2019).

A key example is the lysosomal mTORC1–TFEB/TFE3 axis. Amino-acid availability is sensed through lysosome-associated machinery, which allows mTORC1 to be recruited to and activated at the lysosomal surface (Sancak et al., 2010). Once activated, mTORC1 promotes anabolic growth and suppresses autophagy and lysosomal biogenesis by retaining TFEB and TFE3 in the cytoplasm through phosphorylation-dependent mechanisms (Chen et al., 2024; Martina et al., 2014; Settembre et al., 2012). During starvation or lysosomal stress, reduced mTORC1 activity allows TFEB and TFE3 to translocate into the nucleus, thereby initiating a transcriptional program that drives autophagy and lysosome renewal (Sardiello et al., 2009). Cui et al. further resolved the structure of the lysosomal mTORC1–TFEB–Rag–Ragulator megacomplex, providing molecular-level evidence for how TFEB is presented to mTORC1 for phosphorylation (Cui et al., 2023b).

This membrane-based signaling architecture also implies that lysosomes are intrinsically heterogeneous, with their signaling and degradative states varying according to nutrient availability, vesicle traffic and cellular context (Bond et al., 2025; Savini et al., 2019). Late endosomes, endolysosomes, autolysosomes and phagolysosomes differ in membrane protein composition, Rab-dependent trafficking status, luminal pH, hydrolase activity, ion-channel activity and cargo burden (Kumar et al., 2024). Therefore, LAMP1 positivity alone is insufficient to define a compartment as a functional lysosome, particularly in neurons and other CNS cells where LAMP1-positive organelles may represent diverse degradative or pre-degradative compartments (Cheng et al., 2018). A LAMP1-positive compartment may be enlarged but poorly acidified, hydrolase-containing but fusion-incompetent, or mTORC1-positive yet catabolically insufficient (Quick et al., 2023).

This distinction is particularly important in studies of CNS injury and neuroinflammation. In these cases, lysosomal changes usually reflect the combined effects of phagocytic load, inflammatory activation and degradation stress, rather than mere lysosome function enhancement (Hegdekar et al., 2023; Quick et al., 2023). After spinal cord injury, microglia and infiltrating macrophages must process a large amount of myelin debris and damaged cells. However, this increased lysosomal demand may exceed their ability to acidify lysosomes, mature hydrolases, and complete phagolysosomal degradation (Li et al., 2022). Similarly, the accumulation of LC3-positive autophagic structures in neurons may reflect the downstream blocking of autophagic flux, rather than enhanced autophagy initiation. This blockage includes autophagosome–lysosome fusion defects or insufficient lysosomal degradation ability (Lang et al., 2024; Nixon and Rubinsztein, 2024). Thus, lysosomal remodeling should be understood as a functional state. It integrates acidification, fusion ability, cargo degradation and inflammatory output, and should not be regarded as a phenotype defined by a single marker (Lei et al., 2024; Yang and Tan, 2023).

### 2.2 Luminal acidification and hydrolase activation

The acidic lysosomal lumen provides a chemical basis for intracellular degradation. In most mammalian cells, the lysosomal pH is maintained at about 4.5–5.0, mainly depending on the activity of V-ATPase. The enzyme hydrolyzes adenosine triphosphate (ATP) and drives proton transport into the lysosomal lumen. This proton gradient is not merely a biochemical feature of lysosomes; it can promote the maturation and catalytic activity of acid hydrolases and maintain the luminal environment required for the degradation of proteins, lipids, glycans, and nucleic acids (Chen Y. Y. et al., 2025; Colacurcio and Nixon, 2016; Mindell, 2012; Song et al., 2020). Therefore, V-ATPase-dependent acidification is a proximal factor that determines the degradation ability of lysosomes.

However, lysosomal acidification is not only determined by proton pumps. Because the proton transport mediated by V-ATPase is electrogenic, the proton accumulation in the lumen must be balanced by counter-ion movement to maintain membrane potential and pH stability. Chloride ion transport, cation conductance and proton leakage pathways together shape the electrochemical state in the lysosomal lumen (DiCiccio and Steinberg, 2011; Stauber and Jentsch, 2013; Xu and Ren, 2015). A particularly important example is chloride channel 7 (ClC-7). It is a lysosomal 2Cl^-^/H^+^ exchanger which uses the proton gradient generated by V-ATPase to promote the accumulation of chloride ions in the phagolysosomes. This chloride-rich environment supports the activity of a variety of lysosomal resident hydrolases and promotes phagosome maturation and resolution (Coppola et al., 2023; Feng et al., 2023; Wu et al., 2023).

Therefore, hydrolase activation is inseparable from an acidic and ionically suitable lysosomal lumen. Many lysosomal hydrolases, including cathepsins, are delivered in the form of inactive precursors and need to be processed under acidic conditions to obtain mature enzyme activity (Appelqvist et al., 2013). Correspondingly, even if lysosomes contain large amounts of hydrolase proteins, their function may still be impaired if luminal acidification is insufficient (Lee et al., 2022). When interpreting lysosomal markers, this distinction is very important: the increase in cathepsin D, cathepsin B or LAMP-related signals should not be directly regarded as evidence of enhanced degradation, unless they are accompanied by pH recovery, substrate cleavage enhancement, and restored autophagic or phagolysosomal flux (Klionsky et al., 2021; Mauvezin and Neufeld, 2015).

In pathological states of the CNS, the failure of lysosomal acidification may transform lysosomes from degradative compartments into sites of cargo retention and cellular stress (Colacurcio and Nixon, 2016). In neurons, acidification defects in autolysosomes may impair the removal of damaged mitochondria, autophagic substrates and aggregate proteins. This mechanism has been highlighted in Alzheimer’s disease models, where neuronal autolysosome de-acidification and reduced V-ATPase activity occur before overt extracellular amyloid deposition and are associated with intraneuronal accumulation of amyloid-β/amyloid precursor protein β-C-terminal fragment-positive (Aβ/APP-βCTF) autophagic vacuoles (Im et al., 2023; Lee et al., 2022; Lin et al., 2025). In microglia and infiltrating macrophages, insufficient lysosomal acidification may weaken both phagocytic and autophagic degradation. This can delay the digestion of myelin debris, apoptotic material and inflammatory cargo, thereby sustaining neuroinflammatory signaling. Defective microglial acidification has been linked to impaired phagocytic and autophagic function and to the persistence of neuroinflammation in neurodegenerative contexts (Berglund et al., 2020; Hegdekar et al., 2023; Majumdar et al., 2007). In oligodendrocyte-lineage cells and demyelinating lesions, impaired lysosomal degradation may further disturb lipid turnover, membrane remodeling and remyelination, because effective repair requires not only myelin uptake but also lysosomal lipid processing and metabolic adaptation (Cantuti-Castelvetri et al., 2018; Chen H. et al., 2025; Cignarella et al., 2020).

Thus, lysosomal pH should not be viewed as merely a technical readout. It is a functional determinant that affects whether CNS cells remove damage-related cargo or transform lysosomal load into persistent inflammation, debris removal defects and repair failure (Zeng et al., 2023; Zhang J. et al., 2023). For this reason, lysosomal acidification should be evaluated by ratiometric pH probes, hydrolase activity detection, substrate degradation reporting system and flux-based autophagy detection, and should not be inferred solely from lysosome abundance (Klionsky et al., 2021; Solé-Domènech et al., 2025; Yao et al., 2023).

### 2.3 Ionic and redox regulation in lysosomes

Lysosomes maintain steep ionic gradients across their limiting membrane. Beyond protons, Ca^2+^, Na^+^, K^+^, Cl^−^, Fe^2+^/Fe^3+^, Zn^2+^ and Cu^+^/Cu^2+^ collectively shape lysosomal signaling, trafficking, fusion competence, pH regulation and redox balance (Kondratskyi et al., 2025). This ionic organization allows lysosomes to function as local signaling organelles, rather than as insulated digestive vacuoles (Hu et al., 2024). Recent reviews of endomembrane ion channels and lysosomal transporters have therefore placed lysosomal ion fluxes at the center of organelle communication, stress adaptation and disease-relevant signaling responses (Kondratskyi et al., 2025).

Lysosomal Ca^2+^ is particularly important in this context. Transient receptor potential mucolipin 1 (TRPML1)-mediated Ca^2+^ release regulates vesicular trafficking, autophagosome–lysosome fusion, lysosome reformation and TFEB-dependent lysosomal gene programs (Medina et al., 2015). Experimental work has shown that TRPML1 connects lysosomal Ca^2+^ signaling with rapid autophagic remodeling and longer-term lysosomal activation (Scotto Rosato et al., 2019). More studies suggest that lysosomal Ca^2+^ signaling can link mitochondrial or metabolic stress to TFEB activation, mitophagy and adaptive lysosomal biogenesis (Bhattacharjee et al., 2024; Park and Lee, 2022).

Redox-active metals add another layer of regulation to lysosomal chemistry. Lysosomes are an important site for iron recycling, because ferritin, mitochondria and other iron-containing substrates can be delivered to the lysosomal compartment for degradation. The process is strictly controlled by nuclear receptor coactivator 4 (NCOA4)-mediated ferritinophagy and other mechanisms, thus linking selective autophagy with intracellular iron mobilization and iron homeostasis (Hoelzgen et al., 2024; Mancias et al., 2014). Under physiological conditions, lysosomal iron recycling supports metabolic enzyme activity, mitochondrial function and cellular iron homeostasis. However, under stress conditions, excessive lysosomal Fe^2+^ may drive the Fenton reaction and lipid peroxidation. Recent studies have shown that lysosomal iron activation is sufficient to trigger membrane lipid oxidation and ferroptosis, suggesting that lysosomes are not passive iron storage compartments, but rather active sites of iron-dependent oxidative cell death (Cañeque et al., 2025).

This chemistry is highly relevant to CNS injury. Spinal cord injury, traumatic brain injury, and demyelinating disease expose phagocytes and neural cells to myelin lipids, heme-derived products, damaged mitochondria and inflammatory oxidants (Gao C. et al., 2023; Lloyd and Miron, 2019). When lysosomal acidification, Ca^2+^-dependent membrane dynamics, metal handling and membrane stability are preserved, these substrates can be degraded and recycled through autophagic and phagolysosomal pathways (Nixon and Rubinsztein, 2024; Radulovic et al., 2026). When this control fails, lysosomes may instead become local amplifiers of oxidative injury, ferroptosis-like damage and inflammatory activation (Cañeque et al., 2025; Wei et al., 2024). This concept is supported by recent work linking microglial lysosomal membrane proteins to iron deposition and ferroptosis after spinal cord injury (Ouyang et al., 2025). It is also supported by traumatic brain injury studies emphasizing iron metabolism and ferroptosis in secondary injury, and by demyelination studies showing that defective myelin lipid and cholesterol handling can impair repair.

### 2.4 Lysosomal membrane integrity and quality control

Because lysosomes contain acidic hydrolases, cations and redox-active substrates, the integrity of the lysosomal limiting membrane is essential for cell survival. Lysosomal membrane permeabilization can release lysosomal hydrolases, protons, Ca^2+^ and metal ions into the cytosol, thereby linking organelle damage to cell death, inflammasome activation and innate immune signaling (Meyer and Kravic, 2024; Xie et al., 2023). Accordingly, lysosomal membrane homeostasis is maintained through a coordinated quality-control network that balances rapid membrane repair, selective removal of damaged lysosomes and compensatory lysosome regeneration (Meyer and Kravic, 2024; Yang and Tan, 2023).

Mild lysosomal damage can be repaired before extensive leakage occurs. One important mechanism is the phosphoinositide-initiated membrane tethering and lipid transport (PITT) pathway. Following lysosomal membrane permeabilization, phosphatidylinositol 4-kinase type 2 alpha (PI4K2A) is rapidly recruited to damaged lysosomes and generates phosphatidylinositol 4-phosphate (PI4P), which promotes endoplasmic reticulum (ER)–lysosome membrane contacts and lipid transfer to reseal the injured membrane (Tan and Finkel, 2022). This establishes rapid membrane repair as a primary defense against lysosomal leakage.

When damage is severe or persistent, cells initiate lysophagy to eliminate compromised lysosomes. In this process, exposed luminal glycans are recognized by cytosolic galectins, followed by ubiquitination of damaged lysosomal membranes and recruitment of selective autophagy receptors that deliver the injured organelle to the autophagy machinery (Yang and Tan, 2023). Recent work further identified spastic paraplegia 20 (SPG20)–ITCH E3 ubiquitin-protein ligase (ITCH) as a damage-sensing module that detects lipid bilayer perturbation and initiates ubiquitin-dependent lysophagy, thereby helping cells triage severely damaged lysosomes for destruction rather than repair (Gahlot et al., 2024).

In CNS injury, microglia and infiltrating macrophages internalize large amounts of myelin debris, apoptotic material and damage-associated molecules, making their function dependent on intact endolysosomal degradation (Hammel et al., 2022). Lysosomal membrane repair and lysophagy are established quality-control mechanisms that reseal mildly damaged lysosomes or selectively remove irreversibly injured lysosomes, thereby preventing hydrolase leakage, ionic disturbance and inflammasome activation. Therefore, in injury-activated phagocytes, intact lysosomal membrane quality control may help preserve degradative capacity and restrain excessive inflammatory activation. Conversely, accumulated lysosomal damage may shift phagocytes from debris-clearing cells toward sustained amplifiers of neuroinflammation and secondary tissue injury (Li et al., 2022; Ma et al., 2025).

## 3 Lysosomes as metabolic recycling hubs in the central nervous system

In the CNS, lysosomes are not merely terminal degradative organelles but metabolic recycling hubs that continuously convert proteins, lipids, glycans and damaged organelles into reusable substrates (Ebner et al., 2025; Freeze et al., 2022; Radulovic et al., 2026). This effect is especially important in post-mitotic neurons, microglia with high phagocytic activity, and metabolically reactive astrocytes. In these cells, cellular homeostasis depends on the balance between cargo delivery, lysosomal degradation, metabolite efflux and downstream nutrient sensing (Bhupana et al., 2025; Sprenger et al., 2025). Recent research has further strengthened this view. These studies show that lysosomes are not only degradation endpoints for endogenous and exogenous macromolecules and damaged organelles but also signaling platforms for metabolic regulatory factors such as mTORC1 (Acharya and Demetriades, 2024).

The functional demands placed on lysosomes differ among CNS cell types (Figure 1). In neurons, lysosomes are required for long-term proteostasis, mitochondrial clearance, and quality control within distal axonal and synaptic compartments (Farfel-Becker et al., 2019). In microglia, they couple the uptake of cellular, proteinaceous, and myelin-derived debris to intracellular digestion and inflammatory resolution (Safaiyan et al., 2016). Astrocytes use lysosomal pathways for endocytic cargo handling and extracellular matrix turnover, thereby contributing to metabolic support, tissue remodeling, and regulation of the local inflammatory environment (Chung et al., 2013). Oligodendrocyte-lineage cells depend particularly on lysosomal lipid turnover and membrane remodeling to maintain myelin integrity and support remyelination (Aber et al., 2022). These functions are not restricted to a single cell type; rather, their relative prominence reflects the specialized physiological roles and cargo burdens of each cell population.

### 3.1 Protein degradation and amino acid recycling

Protein turnover in CNS cells is shaped by both cell longevity and spatial complexity. Neurons must preserve proteostasis across the soma, axons, dendrites and synapses for decades, whereas glial cells rapidly remodel their proteome during inflammation, phagocytosis and injury responses (Gao C. et al., 2023; Karpova et al., 2025). The autophagy–lysosome system degrades long-lived proteins, damaged protein complexes and aggregation-prone substrates through macroautophagy, endolysosomal trafficking and selective autophagy (Nixon and Rubinsztein, 2024; Zhao et al., 2024). After lysosomal proteases cleave these cargos into amino acids and short peptides, the resulting metabolites are exported to the cytosol and reused for protein synthesis, mitochondrial metabolism, redox control and nutrient-sensitive signaling pathways (Acharya and Demetriades, 2024; Cui et al., 2023a). Beyond supporting protein resynthesis and nutrient-sensitive signaling, lysosome-derived amino acids may influence mitochondrial bioenergetics and redox balance, but direct evidence for this link in CNS injury remains limited.

This recycling process is directly connected to lysosomal nutrient sensing, as described in Sections 2.1 and 2.2 (Bar-Peled et al., 2012; Sancak et al., 2010; Zoncu et al., 2011). More recent work further indicates that amino acids derived from different intracellular sources can engage spatially and functionally distinct pools of mTORC1 (Fernandes et al., 2024). Conversely, nutrient limitation, proteotoxic stress or organelle damage will promote autophagic flux so that cells can mobilize internal macromolecular reserves (Liénard et al., 2024; Nixon and Rubinsztein, 2024). This balance is especially important in CNS injury and neurodegenerative diseases, because neurons and glial cells must simultaneously remove damaged proteins, adapt to inflammatory stress, and maintain energy production (Quick et al., 2023; Wang B. et al., 2024). Overall, these findings support a model in which lysosomal protein recycling coordinates proteostasis, inflammatory adaptation and metabolic resilience in the damaged or degenerating CNS.

Because axons and synapses are far from the cell body, even mild defects in autophagosome formation and maturation, axonal endolysosomal transport or autophagosome–lysosome fusion may lead to local accumulation of damaged proteins, synaptic components and dysfunctional organelles (Karpova et al., 2025; Roney et al., 2022). This local disorder may interfere with synaptic vesicle cycling, cytoskeletal stability and axonal transport before the obvious loss of neurons occurs (De Pace et al., 2024). Therefore, neuronal lysosomes are not only involved in general protein degradation, but also in the compartment-specific quality control in axons, dendrites and synapses. These observations suggest that neuronal lysosomal dysfunction may first damage local protein homeostasis and axonal–synaptic integrity, and then further converge into broader neurodegenerative changes. However, the timing and order of these events may vary depending on disease context.

In microglia, lysosomal protein degradation is closely related to phagocytic function. As resident immune phagocytic cells in the CNS, microglia continuously monitor the brain parenchyma and remove synaptic components, apoptotic fragments, protein aggregates and debris produced by injury through phagocytic and endolysosomal pathways (Choi and Chung, 2025; Gao C. et al., 2023). The engulfed cargo must be efficiently delivered to lysosomes and degraded in the acidified degradative compartments; otherwise, phagocytosis may be decoupled from the complete cargo clearance process. If this process is incomplete, the internalized cargo may persist in the endolysosomal compartment, thus maintaining inflammatory signaling and promoting the formation of disease-related microglial phenotypes (Quick et al., 2023; Scheiblich et al., 2024; Wang B. et al., 2024). Therefore, lysosomal protein degradation in microglia can be regarded as a functional checkpoint. It links phagocytic uptake with two different outcomes: one is the cargo clearance and repair-oriented remodeling, and the other is the activation of persistent inflammation.

### 3.2 Lipid turnover, cholesterol trafficking and myelin debris degradation

The CNS carries a particularly high lipid burden because myelin is enriched in cholesterol, phospholipids and sphingolipids. Lysosomes process endocytosed lipoproteins, membrane fragments, autophagic lipid cargo and phagocytosed myelin debris, after which cholesterol and other lipid species are redirected for membrane repair, lipid-droplet storage or extracellular efflux (Wuerch and Yong, 2025). Under physiological conditions, this lipid-recycling system supports membrane renewal and myelin homeostasis. Under pathological conditions, however, demyelination, axonal degeneration and tissue injury sharply increase the influx of lipid-rich debris, placing a heavy metabolic burden on microglia and infiltrating macrophages. When cholesterol processing or lipid efflux is impaired, lipid-loaded phagocytes may sustain inflammation and delay repair (Montilla et al., 2025; Zhao et al., 2025).

A key point is that myelin uptake and myelin degradation are separable events. Microglia may engulf myelin debris efficiently, yet still fail to process the internalized lipid load when lysosomal acidification, cholesterol export or lipid trafficking is impaired (Colombo et al., 2021; Montilla et al., 2025). In this setting, phagocytosed cargo accumulates within endolysosomal compartments, cholesterol can become trapped in lysosomes, and excess lipids may be redirected into lipid droplets. This mismatch may push microglia away from a debris-clearing state and toward a lipid-stressed, inflammatory phenotype (Haney et al., 2024; Yu J. B. et al., 2025). Thus, the functional outcome of myelin phagocytosis depends less on uptake itself than on whether lysosomes can complete lipid digestion and cholesterol redistribution.

This concept is relevant to demyelinating disease, aging and neurodegeneration. In Alzheimer’s disease, microglial states associated with apolipoprotein E (APOE) and phosphatidylinositol-binding clathrin assembly protein (PICALM) have been linked to cholesterol-rich lysosomes, lipid-droplet accumulation, oxidative stress and impaired phagocytosis (Haney et al., 2024; Kozlova et al., 2025). In demyelinating lesions, inefficient clearance of myelin-derived lipids may delay remyelination, because persistent myelin debris inhibits oligodendrocyte precursor differentiation and maintains an inflammatory lesion environment (Kotter et al., 2006; Montilla et al., 2025). Conversely, by removing inhibitory myelin debris, restoring cholesterol homeostasis and supporting vascular remodeling and tissue homeostasis, lipid-responsive microglia and macrophages may support repair when lysosomal degradation and cholesterol-export function remain intact (Berghoff et al., 2021).

Thus, lysosomal lipid turnover should be regarded as a metabolic checkpoint, not a simple degradative pathway. Efficient lipid degradation helps debris removal and tissue repair. By contrast, cholesterol transport defects may turn the same phagocytic process into a driver of inflammation and degenerative changes. This distinction is especially important in CNS injury, because there are a large number of lipid-rich myelin sheath fragments, and the balance between repair-associated and dysfunctional phagocyte states may affect long-term functional recovery.

### 3.3 Glycan degradation, extracellular matrix remodeling and lysosome–mitochondria crosstalk

Beyond proteins and lipids, lysosomes also degrade glycans, glycoproteins, proteoglycans and extracellular matrix-derived cargos. In the CNS, this function is important because the extracellular matrix affects synapses, axon growth, perineuronal nets, glial activation, and scar formation (Tewari et al., 2022; Tewari et al., 2024). Internalized matrix components can be routed to lysosomes and degraded by glycosidases, sulfatases and proteases, linking lysosomal catabolism to the turnover of glycan-rich extracellular structures (Baudot et al., 2022; Nguyen et al., 2020). In parallel, lysosomal exocytosis may release selected hydrolases into the extracellular space, where they can contribute to local matrix remodeling (Trojani et al., 2024). In CNS injury, this mechanism may provide a lysosome-dependent route through which cells couple intracellular cargo clearance to extracellular matrix reorganization.

After spinal cord injury, traumatic brain injury, stroke or demyelination, extracellular matrix remodeling is required for immune-cell entry, debris clearance, angiogenesis, glial scar maturation and axonal plasticity (Li H. et al., 2023; Tewari et al., 2022). However, this process itself has context-dependent effects. Controlled matrix turnover may promote tissue reorganization and repair. On the contrary, excessive or disordered degradation may destroy tissue structure stability, expose damage-related signals, and amplify inflammatory reactions (Li H. et al., 2023). Therefore, lysosome-dependent matrix treatment should be regarded as a context-dependent mechanism. It can support tissue reconstruction but may also participate in pathological remodeling.

Another level of lysosomal recycling is organelle quality control, especially mitochondrial turnover. Mitochondria produce ATP, buffer calcium ions, and provide metabolic intermediates. Lysosomes remove dysfunctional mitochondria through mitophagy and recover their components to support metabolic recovery (Uoselis et al., 2023; Wang et al., 2023). This lysosome-mitochondria axis is particularly important in CNS cells: neurons need to continuously obtain local energy supply along the axons and synapses; microglia undergo rapid metabolic conversion during activation; oligodendrocytes rely on coordinated mitochondrial and lipid metabolism to maintain myelin formation and stability (Asadollahi et al., 2024; Bame and Hill, 2024; Liang et al., 2024; Wang H. et al., 2024).

In neurons, mitochondrial autophagy defects may cause damaged mitochondria to stay in axons and synapses, resulting in local ATP deficiency, disrupted calcium homeostasis and oxidative injury (Li J. et al., 2023; Liang et al., 2024). In microglia, insufficient mitochondrial clearance may enhance mitochondrial reactive oxygen species (ROS) production and innate immune signaling, thus further amplifying inflammatory activation (Shokr, 2025; Wang H. et al., 2024). Conversely, well-preserved lysosomal function can support mitochondrial quality control during phagocytosis and help microglia adapt to increased metabolic needs (Fairley et al., 2023; Zou et al., 2026). Thus, lysosomes are not just about removing damaged organelles. They also help to determine whether mitochondrial stress is ultimately eliminated, recycled, or converted into inflammatory signals.

In general, lysosomal recycling in CNS cells integrates protein degradation, lipid turnover, glycan degradation, matrix handling and organelle quality control. These processes are not isolated pathways, but interconnected layers of metabolic adaptation. They link the degradation of cargo with nutrient sensing, inflammatory regulation and cellular stress responses (De Tito and Tooze, 2026; Ferrari et al., 2024). When lysosomal recycling can match cellular needs, it supports protein homeostasis, debris removal, tissue remodeling and bioenergetic recovery (Ebner et al., 2025; Quick et al., 2023). When this coordination fails, the same recycling system may turn into a source of proteotoxicity, lipid stress, inflammatory activation and neurodegenerative changes (Tsap and Shcherbata, 2025; Udayar et al., 2022). Thus, lysosomal recycling should not be regarded as a set of parallel degradation routes, but should be understood as an integrated metabolic checkpoint. It determines whether CNS cells adapt to injury or enter a maladaptive state. Although CNS cells share the same core lysosomal machinery, its functional emphasis differs by cell type, supporting proteostasis and axonal quality control in neurons, debris clearance and inflammatory regulation in microglia, cargo handling and matrix remodeling in astrocytes, and lipid turnover and membrane adaptation in oligodendrocyte-lineage cells (Figure 1). Accordingly, similar lysosomal defects may produce distinct pathological outcomes depending on the affected cell type and accumulated cargo, providing a basis for the disease-specific remodeling discussed below.

## 4 Pathological lysosomal remodeling in CNS disorders

In diseased CNS cells, lysosomes are not passive degradation endpoints. Rather, they may initially undergo adaptive remodeling; when the cargo burden exceeds their capacity, they become dysfunctional, thereby affecting proteostasis, lipid processing, immune activation and cell fate. In acute injury, this shift is driven by the sudden accumulation of damaged organelles, necrotic debris, myelin-derived lipids and blood-derived redox-active substrates, all of which contribute to secondary tissue injury and impose heavy degradative and metabolic burden on neural and immune cells (Hu et al., 2023; Li et al., 2026b). In chronic neurodegeneration, this remodeling is sustained by misfolded proteins, lipid dyshomeostasis, mitochondrial stress and aging-related failure of lysosomal repair and proteostatic maintenance (Chou et al., 2025; Palmer et al., 2025). Thus, pathological lysosomal remodeling should not be interpreted as a uniform increase or decrease in degradative activity, but as a context-dependent reprogramming of lysosomal identity and function.

### 4.1 Spinal cord injury

Spinal cord injury (SCI) imposes a dense degradative burden within a confined CNS compartment. Mechanical disruption of axons, myelin, blood vessels and resident neural cells rapidly generates necrotic debris, damaged organelles, extracellular matrix fragments, myelin-derived lipids and blood-derived iron, all of which reshape the inflammatory, metabolic and redox state of the lesion (Hu et al., 2023; Zheng et al., 2025). These substrates impose substantial degradative, metabolic, and redox stress on lysosomes.

Lysosomal remodeling may also depend on the injury model. Contusion produces heterogeneous tissue damage, hemorrhage, and a broad distribution of myelin and cellular debris, whereas transection creates a more sharply defined lesion with abrupt loss of tissue continuity (Choo et al., 2007; Siegenthaler et al., 2007). These differences are likely to alter the amount, composition, and persistence of cargo encountered by neurons and phagocytes (Ryan et al., 2022). However, direct comparisons of lysosomal pH, hydrolase activity, membrane integrity, or lipid flux between SCI models remain limited.

Microglia and macrophages are the principal phagocytes that cope with this lysosomal burden after SCI. Importantly, this reaction itself is not necessarily harmful. The removal of microglia can aggravate tissue damage and affect the recovery of function, which suggests that the outcome of microglial activation depends on whether phagocytic ingestion is coupled with effective lysosomal processing (Brennan et al., 2022). When myelin uptake exceeds intracellular lipid-processing and cholesterol-export capacity, lipid-laden phagocytes may acquire foam-cell-like features, sustain pathological signaling, and restrict tissue repair (Peng et al., 2025).

Hemorrhage and demyelination also increase the amount of iron-containing material that must be processed within the lesion. Phagocytes normally limit iron toxicity by coordinating the degradation of iron-bearing cargo with iron storage and trafficking. When this control is lost, redox-active iron can drive lipid peroxidation, ferroptosis injury, and persistent inflammation (Wang et al., 2025; Zheng et al., 2025).

Neurons experience lysosomal stress in a different way. After axonal injury, they must remove damaged mitochondria, disrupted membranes and misfolded proteins while dealing with energy insufficiency and impaired axonal transport. Autophagy may therefore be activated but cannot be completed efficiently, resulting in the accumulation of autophagic vesicles and incompletely degraded cargo (Gao et al., 2024; Wang et al., 2025). Importantly, the accumulation of microtubule-associated protein 1 light chain 3 (LC3-II) or sequestosome 1 (SQSTM1/p62) alone does not indicate increased autophagic activity (Klionsky et al., 2021). In contusion models, reduced lysosomal hydrolase activity and lysosomal membrane damage occur together with autophagic substrate accumulation, supporting a failure of terminal degradation (Li et al., 2019; Liu et al., 2015). Experimental preservation of lysosomal function improves cargo clearance and reduces neuronal injury, suggesting that lysosomal failure contributes directly to autophagic flux blockade. Astrocytes and oligodendrocytes further expand the lysosomal landscape of SCI. Reactive astrocytes help remodel the extracellular matrix, while oligodendrocytes and oligodendrocyte precursor cells (OPCs) face substantial protein and lipid stress during demyelination and attempted remyelination (Brennan et al., 2022; Li et al., 2026b). Their lysosomal states may directly determine scar architecture, white-matter preservation, and later repair capacity, but this question remains underexplored.

Thus, lysosomes link debris clearance, lipid and iron handling, organelle quality control, and inflammatory resolution after SCI. Current evidence more directly supports impaired hydrolase activity, lysosomal membrane damage, and failure of terminal cargo degradation than a primary defect in lysosomal acidification. Future studies should therefore combine lysosomal pH measurements with cathepsin maturation, membrane-damage reporters, autophagic flux, lipid turnover, and lysosomal biogenesis to distinguish adaptive remodeling from functional failure.

### 4.2 Traumatic brain injury

Traumatic brain injury (TBI) also causes acute lysosomal stress, but its disease course is different from that of SCI. TBI commonly involves diffuse axonal injury, blood–brain barrier damage and persistent cognitive impairment (Armstrong et al., 2024; Zhao and He, 2025). The lysosomal system is not only involved in the removal of early injury-associated debris, but also in the maintenance of proteostasis at later stages.

In neurons, TBI-related lysosomal dysfunction is closely linked to disrupted axonal transport. Autophagosomes can be formed in damaged axons, but when axonal transport is blocked, their retrograde transport to somatic regions may be impaired (Luo et al., 2024; Paumier and Gowrishankar, 2024). As a result, damaged proteins and organelles may continue to stay in axons and synapses, leading to local degeneration and neural network dysfunction.

An important clinical consequence of TBI-related lysosomal dysfunction is the link between it and tau pathology. In a controlled cortical impact model, neuronal loss of BCL2-associated athanogene 3 (BAG3) impaired autophagy–lysosome function and promoted tau hyperphosphorylation, synaptic dysfunction, and cognitive deficits, whereas BAG3 restoration alleviated these changes (Sweeney et al., 2024). These findings link impaired lysosomal proteostasis to post-traumatic neurodegenerative changes.

Glial lysosomes also shape the post-traumatic inflammatory environment. After TBI, microglia and astrocytes are involved in the handling of synaptic fragments, myelin debris and dead cells, but impaired autophagy–lysosome flux may prolong neuroinflammation and secondary injury (Bano et al., 2025; Sarkar and Lipinski, 2024; Zhang et al., 2025). Incomplete lysosomal degradation may sustain inflammasome, complement, and cytokine signaling.

### 4.3 Alzheimer’s disease

Alzheimer’s disease (AD) is a typical chronic disorder in which lysosomal remodeling can be transformed into dysfunction. Although AD is classically characterized by extracellular amyloid-β plaques, intracellular tau tangles, synaptic failure and neuronal loss, more and more evidence shows that the endosomal–lysosomal system is one of the earliest vulnerable cellular compartments, rather than a passive terminal degradative sink (Lee et al., 2022; Nixon and Rubinsztein, 2024). This view is important because it suggests that lysosomal dysfunction may contribute to the initiation and spread of AD pathology, rather than occurring merely as a consequence of amyloid and tau accumulation.

A recent study showed that aging and AD create a cell-autonomous state of neuronal vulnerability, which is characterized by persistent lysosomal damage, impaired protein homeostasis and impaired lysosomal repair (Chou et al., 2025). These abnormalities promoted intraneuronal protein deposition and inflammatory cytokine secretion, indicating that lysosomal damage and its repair failure are upstream factors in neuronal vulnerability in AD.

In neurons, AD-related lysosomal remodeling is not a single defect but an interrelated imbalance between endosomal trafficking, autophagic clearance and lysosomal integrity. Enlarged early endosomes can disturb APP sorting and promote amyloidogenic processing, while impaired autophagy–lysosome clearance allows abnormal proteins and damaged organelles to persist (Yu Y. et al., 2025; Zadka et al., 2024). More direct evidence places defective acidification upstream of cargo accumulation. In five AD mouse models carrying a neuron-specific tandem fluorescent LC3 and pH reporter, poorly acidified autolysosomes and reduced V-ATPase activity were detected before extensive extracellular amyloid deposition. These compartments selectively accumulated APP-βCTF and Aβ (Lee et al., 2022). Mechanistically, Tyr682-phosphorylated APP-βCTF can bind V-ATPase V0 subunit a1 (V0a1) and interfere with V1–V0 assembly of V-ATPase. Reducing APP-βCTF phosphorylation restored lysosomal acidification and degradative function in Down syndrome and AD models (Im et al., 2023). However, the upstream cause of defective acidification remains debated. Earlier studies linked presenilin 1 deficiency to impaired V0a1 trafficking, reduced V-ATPase activity, and defective lysosomal acidification (Lee et al., 2010), whereas subsequent work did not reproduce a general presenilin-dependent defect in autophagic clearance (Zhang et al., 2012). Acidification failure should therefore be viewed as a mechanism that varies with genotype, cell type, and disease stage rather than as a uniform feature of AD. When lysosomal membranes are injured, hydrolases, Ca^2+^ and other stress signals may leak into the cytosol, further weakening the protein homeostasis and organelle quality control of neurons (Ferrari et al., 2024).

Microglial lysosomes are also involved in disease regulation. Around amyloid plaques, microglia alter phagocytosis, lipid processing and lysosomal gene expression; however, this reaction has a protective effect only when effective degradation can be completed after ingestion (Wang B. et al., 2024; Wu et al., 2025). Disruption of TFEB–V-ATPase signaling impairs lysosomal homeostasis and changes the activation state of microglia in the context of tauopathy and AD, which suggests that microglial activation alone is insufficient; the key factor is whether lysosomes can complete cargo processing after phagocytosis (Wang B. et al., 2024). In line with this, reducing the lipid load of microglia in the AD mouse model enhanced β-amyloid clearance, which supports the view that lysosomes under lipid stress may limit the degradation ability of microglia (Wu et al., 2025). Microglial lysosomal capacity may therefore determine whether plaque-associated activation remains protective or becomes dysfunctional.

AD-related lysosomal pathology can be understood from three interdependent levels. In neurons, lysosomal damage and repair defects can promote intracellular proteotoxic stress and weaken organelle quality control (Chou et al., 2025). At the level of microglia, excessive phagolysosomal stress and lipid accumulation will weaken the processing of amyloid- and tau-related cargo and reshape the immune response (Wang B. et al., 2024; Wu et al., 2025). At the tissue level, amyloid, tau, disrupted lipid homeostasis, complement activation and cytokine signaling reinforce each other, forming a chronic and intractable degenerative microenvironment (Heneka et al., 2025; Kuhn and Proctor, 2025). This framework may explain why removing extracellular amyloid alone does not necessarily restore the homeostasis of neurons and glial cells, unless the lysosomal function is also restored.

### 4.4 Parkinson’s disease

In Parkinson’s disease (PD), accumulation of α-synuclein, the vulnerability of dopaminergic neurons and a variety of PD risk genes all converge on autophagy–lysosome dysfunction (Bayati and McPherson, 2024; Matsui and Takahashi, 2024). Compared with AD, PD more directly links lysosomal proteolysis to mitochondrial quality control, synaptic vesicle homeostasis and dopamine-related oxidative stress (Giamogante et al., 2024; Mingo et al., 2025).

Multiple PD-related genes directly affect the lysosomal pathway. Glucocerebrosidase beta 1 (GBA1) mutations reduce glucocerebrosidase activity and disturb lysosomal lipid homeostasis (Ayloo et al., 2026; Dai et al., 2024). Leucine-rich repeat kinase 2 (LRRK2) regulates Rab-dependent endolysosomal trafficking and lysosomal membrane remodeling (Bentley-DeSousa et al., 2025; Bonet-Ponce et al., 2024). Other PD genes, including vacuolar protein sorting 35 (VPS35) and ATPase cation transporting 13A2 (ATP13A2), further connect retromer-dependent recycling and ion homeostasis with lysosomal stress (Alessi et al., 2024; Qiu et al., 2024). These findings suggest that lysosomal dysfunction contributes to neuronal vulnerability in both familial and sporadic PD (Dai et al., 2024; Klein and Mazzulli, 2018).

There is a bidirectional relationship between α-synuclein and lysosomes. Impaired macroautophagy, chaperone-mediated autophagy, or endolysosomal degradation can lead to α-synuclein accumulation, whereas misfolded or aggregated α-synuclein further damages lysosomal proteolysis, vesicle trafficking and membrane dynamics (Bayati and McPherson, 2024; Dai et al., 2024). This feedback loop is especially harmful in dopaminergic neurons, which already face dopamine oxidation, mitochondrial stress and high synaptic activity (Brooker et al., 2024; Matsui and Takahashi, 2024).

Lysosome–mitochondria coupling is another key layer of PD pathology. Insufficient lysosomal acidification or hydrolase activity may stall mitophagy at a late stage, resulting in the persistence of dysfunctional mitochondria. α-Synuclein can change lysosomal-mitochondria contacts and Ca^2+^ transport, linking synuclein pathology with impaired mitochondrial quality control and lysosomal stress signals (Giamogante et al., 2024).

Microglia internalize extracellular α-synuclein through the endolysosomal pathway. Efficient degradation may limit its persistence and spread, whereas incomplete degradation can sustain inflammatory activation and neuronal injury (Eo et al., 2024). As in AD, the outcome depends not only on microglial activation but also on effective post-phagocytic lysosomal clearance.

### 4.5 Other neurological disorders

Lysosomal remodeling is also involved in other neurological disorders, including multiple sclerosis (MS), amyotrophic lateral sclerosis/frontotemporal dementia (ALS/FTD), ischemic brain injury and Huntington’s disease. Although these diseases arise from different initiating insults, they share a common vulnerability: disease-relevant cargo accumulates when lysosomal delivery, degradation, recycling or repair is insufficient (Beckers and Van Damme, 2025; Berg et al., 2025; Nixon and Rubinsztein, 2024).

In demyelinating disease, microglia and macrophages must process lipid-rich myelin debris through lysosomal lipid hydrolysis, cholesterol handling and lipid efflux, thereby influencing inflammatory resolution and remyelination (Montilla et al., 2025; Wuerch and Yong, 2025). In ALS/FTD, chromosome 9 open reading frame 72 (C9orf72), TANK-binding kinase 1 (TBK1), optineurin (OPTN), sequestosome 1 (SQSTM1/p62), and valosin-containing protein (VCP) link selective autophagy, aggregate clearance, vesicular trafficking and innate immune signaling, making motor neurons particularly sensitive to impaired autophagy–lysosome flux (Beckers and Van Damme, 2025; Diab et al., 2023). In ischemic brain injury, lysosomal Ca^2+^ signaling and TFEB activation can support neuronal stress adaptation by restoring autophagic–lysosomal function. In Huntington’s disease, mutant huntingtin disrupts autophagy–lysosome function and lysosomal quality control, contributing to defective protein clearance and neuronal vulnerability (Berg et al., 2025; Luo and Yang, 2024; Rusmini et al., 2025). Together, these disorders illustrate a shared principle: lysosomal remodeling may initially represent an adaptive response, but becomes pathological when degradative capacity, membrane repair and cargo recycling can no longer match the intensity or persistence of disease-associated stress.

### 4.6 Comparative features of lysosomal dysfunction across CNS disorders

Across CNS disorders, lysosomal dysfunction shares several recurring features. In both acute injury and chronic neurodegeneration, the amount and complexity of pathological cargo can exceed lysosomal processing capacity, leading to impaired acidification, reduced hydrolase activity, incomplete autophagic or phagolysosomal flux, defective membrane quality control, and insufficient metabolic recycling. These abnormalities ultimately converge on inefficient clearance of damaged proteins, organelles, lipids, and cellular debris, thereby sustaining oxidative stress, inflammatory signaling, and cellular dysfunction (Li et al., 2019; Nixon and Rubinsztein, 2024; Zheng et al., 2023). Thus, a common pathological principle across CNS diseases is not simply an increase or decrease in lysosome number, but a mismatch between cargo burden and functional degradative competence.

The dominant lysosomal defect nevertheless differs among diseases. In SCI, extensive myelin destruction, hemorrhage, and tissue fragmentation place particular pressure on lysosomal lipid processing, cholesterol redistribution, phagolysosomal degradation, and iron-related redox control in microglia and macrophages (Kroner et al., 2014). TBI also produces an acute degradative burden, but its lysosomal pathology is more strongly associated with disrupted axonal transport, incomplete autophagic flux, delayed tau-related proteotoxicity, and persistent glial inflammation (Sarkar et al., 2014; Sweeney et al., 2024). In AD, endosomal trafficking defects and impaired autolysosomal acidification are prominent early abnormalities that favor the accumulation of Aβ-, APP-βCTF-, and tau-containing cargo, while microglial lipid overload further limits clearance capacity (Lee et al., 2022). In PD, impaired lysosomal proteolysis is closely linked to α-synuclein accumulation, defective mitophagy, and abnormalities in lipid metabolism and vesicular trafficking, particularly in the context of GBA1 and LRRK2 related dysfunction (Eguchi et al., 2018; Mazzulli et al., 2011). Among other neurological disorders, MS is characterized by a pronounced requirement for myelin lipid degradation and cholesterol efflux during remyelination, whereas ALS/FTD and Huntington’s disease more prominently involve defects in selective autophagy, aggregate clearance, endolysosomal trafficking, and lysosomal quality control (Beckers et al., 2023; Martinez-Vicente et al., 2010). These disease-predominant patterns are overlapping rather than mutually exclusive, but they indicate that lysosome-oriented therapy should target the rate-limiting functional module in each pathological context.

## 5 Lysosome-oriented therapeutic strategies for neural repair

Lysosome-oriented therapy is emerging as a mechanistically grounded strategy for neural repair, because lysosomes lie at the intersection of proteostasis, lipid recycling, inflammatory signaling, membrane repair and organelle quality control (Nixon and Rubinsztein, 2024). As summarized in Figure 2, current strategies can be grouped into five categories: restoration of lysosomal acidification and hydrolase activity, activation of TFEB/TFE3-mediated lysosomal biogenesis, regulation of lysosomal ion channels and membrane quality control, reprogramming of lipid turnover and myelin debris clearance, and lysosome-responsive delivery systems. These approaches include small molecules, gene-delivery platforms, signaling regulators, and biomaterials, and they differ considerably in their level of experimental validation. Although several approaches have improved lysosomal or neurological phenotypes in preclinical models, most remain at the proof-of-concept stage, with therapeutic windows, cell-type specificity and *in vivo* target engagement in traumatic CNS injury still insufficiently defined (Chung et al., 2019; Vest et al., 2022).

**FIGURE 2:**
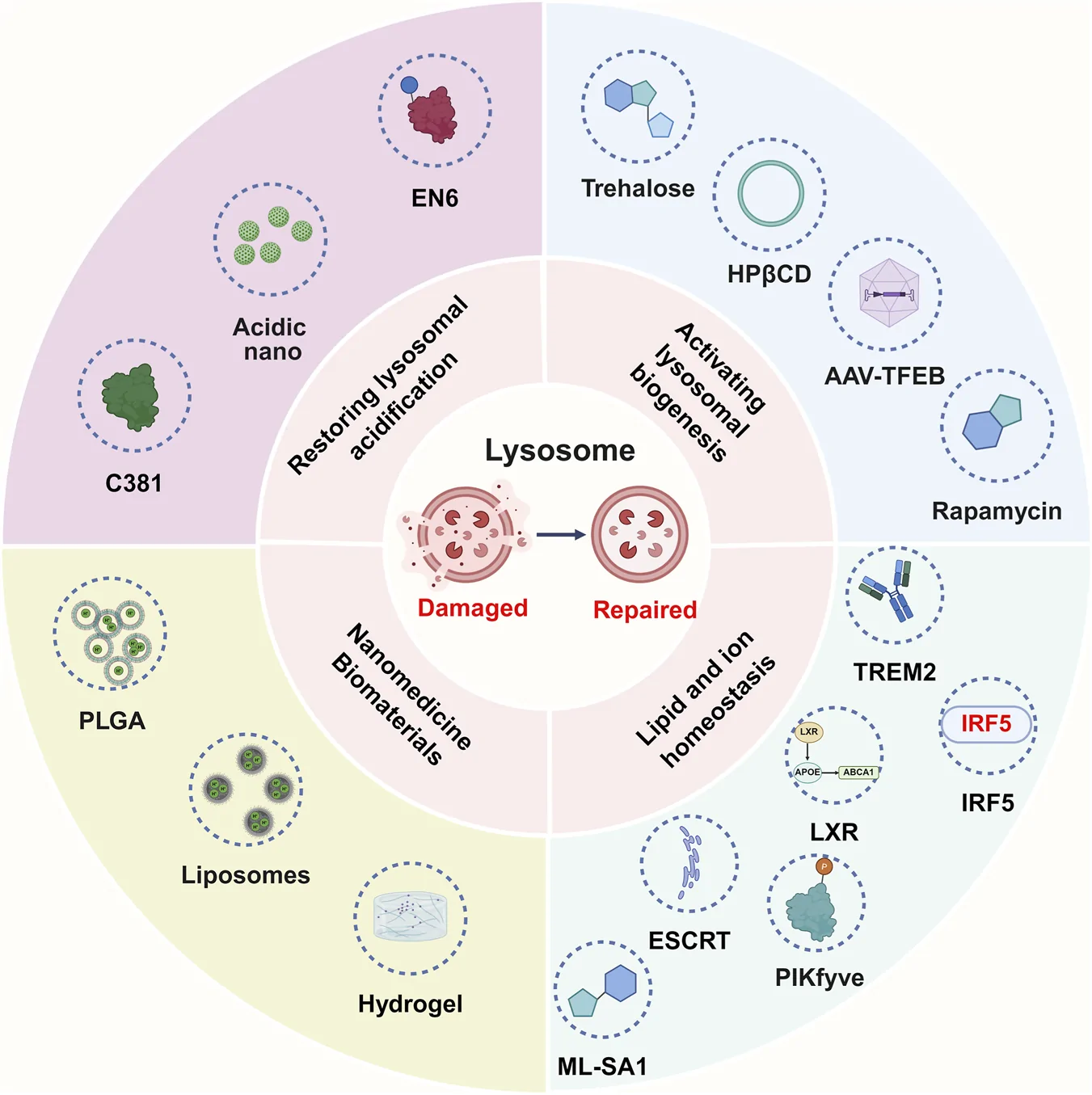
Lysosome-oriented therapeutic strategies for central nervous system injury and repair. Lysosome-oriented therapeutic strategies include restoring lysosomal acidification, activating lysosomal biogenesis, targeting lysosomal ion channels and membrane repair, reprogramming lipid turnover, and developing lysosome-responsive nanomedicine and biomaterials. C381 and EN6 enhance lysosomal acidification through V-ATPase-related mechanisms, whereas acidic nanoparticles directly lower lysosomal pH; trehalose, HPβCD, AAV-TFEB and rapamycin enhance TFEB-related autophagy–lysosome programs; ML-SA1, TRPML1, PIKfyve/PI(3,5)P_2_, ESCRT and lysophagy regulate lysosomal Ca^2+^ signaling, membrane integrity and quality control; LXR–APOE–ABCA1 and TREM2-related pathways promote lipid export and debris clearance; and liposomes, PLGA nanoparticles and hydrogels enable lysosome-targeted delivery and local modulation of lysosomal function. Abbreviations: V-ATPase, vacuolar H^+^-ATPase; HPβCD, 2-hydroxypropyl-β-cyclodextrin; AAV, adeno-associated virus; TFEB, transcription factor EB; ML-SA1, mucolipin synthetic agonist 1; TRPML1, transient receptor potential mucolipin 1; PIKfyve, phosphoinositide kinase, FYVE-type zinc finger containing; PI(3,5)P_2_, phosphatidylinositol 3,5-bisphosphate; ESCRT, endosomal sorting complexes required for transport; LXR, liver X receptor; APOE, apolipoprotein E; ABCA1, ATP-binding cassette transporter A1; TREM2, triggering receptor expressed on myeloid cells 2; PLGA, poly (lactic-co-glycolic acid). Created with BioRender.com, License Number: AT29RG9C22.

### 5.1 Restoring lysosomal acidification and hydrolase activity

#### 5.1.1 C381

C381 is a representative small molecule that restores V-ATPase-dependent proton pumping, as described in Section 2.2. It has been reported to promote lysosomal acidification, enhance lysosomal proteolysis and reduce inflammatory pathology in progranulin-deficient and neurodegeneration-related models (Vest et al., 2022). This approach is most relevant when lysosomal alkalinization, impaired cathepsin activity and autophagic substrate accumulation occur together, because it targets the upstream chemical defect that restricts hydrolase maturation and degradative flux. In CNS injury or neuroinflammatory settings, C381-like lysosomal re-acidification may be particularly useful in phagocytes with high debris burden, but this remains to be directly tested in spinal cord injury models.

#### 5.1.2 EN6

Another pharmacological route is to modulate the V-ATPase machinery itself. EN6 covalently targets the ATPase H^+^ transporting V1 subunit A (ATP6V1A) of lysosomal V-ATPase and has been shown to increase lysosomal acidification, inhibit mTORC1 signaling and activate autophagy (Chung et al., 2019). Compared with broad autophagy inducers, this strategy is conceptually more precise because it acts on the proton-pumping apparatus rather than simply increasing autophagosome formation. Its therapeutic value would be highest in diseases where lysosomal pH elevation is a measurable driver of defective proteolysis, rather than a secondary consequence of terminal cell injury. However, because EN6 evidence is still largely tool-compound based, its translational relevance for CNS repair requires validation in disease-relevant neurons, microglia and infiltrating macrophages.

#### 5.1.3 Acidic nanoparticles

Lysosome-acidifying nanoparticles provide a non-genetic way to correct lysosomal alkalinization. Earlier work showed that acidic nanoparticles can traffic to lysosomes and restore acidic pH, proteolysis, autophagic flux and calcium homeostasis in presenilin-related lysosomal dysfunction models. More recent studies further developed acid-activated or tetrafluorosuccinic acid (TFSA)-based nanoparticles that are preferentially activated in dysfunctional lysosomes, where they release acidic components and restore lysosomal acidification, cathepsin activity, autophagic flux and mitochondrial function (Lo et al., 2024; Zeng et al., 2023). This strategy is attractive because it bypasses some upstream genetic heterogeneity and directly manipulates the lysosomal chemical microenvironment. For neural repair, the key unresolved issue is whether such nanoparticles can be delivered safely and selectively to CNS phagocytes or neurons after injury.

#### 5.1.4 β2-adrenergic agonists

A complementary strategy is to restore lysosomal acidification indirectly by correcting ion-coupled organelle trafficking. In presenilin 1 (PSEN1)-deficient cells and fibroblasts from familial AD patients, β2-adrenergic agonists such as isoproterenol were reported to restore ER-to-lysosome delivery of ClC-7, thereby re-acidifying lysosomes and rescuing lysosomal proteolysis, calcium homeostasis and autophagic flux (Lee et al., 2015; Lee et al., 2020). However, this effect has not yet been independently validated *in vivo* or in traumatic CNS injury models. Subsequent studies indicate that ClC-7 is important for lysosomal chloride accumulation and hydrolase activity, but that its contribution to organelle acidification is context dependent. ClC-7 deficiency impaired intraphagosomal chloride accumulation, hydrolase activity and phagosome resolution without measurably altering phagolysosomal pH, whereas increased ClC-7 activity may promote lysosomal hyperacidification (Leray et al., 2022; Wu et al., 2023). Therefore, β2-adrenergic agonism should currently be viewed as a PSEN1-deficiency-specific proof of concept rather than a broadly validated lysosome-targeted therapy.

### 5.2 Activating TFEB/TFE3-mediated lysosomal biogenesis

#### 5.2.1 Trehalose

Trehalose is a classical enhancer of the autophagy–lysosome system. Mechanistically, it can trigger TFEB nuclear translocation and increase autophagy–lysosome gene expression, partly through mild lysosomal stress and mTORC1-related signaling changes rather than a single linear pathway (Jeong et al., 2021; Rusmini et al., 2019). Therefore, trehalose is better viewed as a metabolic stress-modulating compound that expands lysosomal and autophagic reserve. It may be useful when cells still retain residual degradative competence but require greater lysosomal capacity to handle protein aggregates or damaged organelles. In CNS injury, this strategy may be more suitable for stressed but viable neurons or glia, rather than cells with terminal lysosomal collapse (Figure 2). Because this response is initiated by a modest rise in lysosomal pH, the dose range that activates TFEB without further impairing lysosomal function remains to be defined in injury-relevant cells (Jeong et al., 2021).

#### 5.2.2 Rapamycin or analogues

Rapamycin and rapalogs target the mTORC1–TFEB/TFE3 axis described in Section 2.1 (Napolitano et al., 2022; Zhou et al., 2024; Zwakenberg et al., 2024). However, this strategy lacks cell-type specificity and may produce broad immunometabolic effects. In neural repair, rapamycin-like interventions may be most appropriate when autophagy initiation and lysosomal biogenesis are both suppressed. Their use after CNS injury should be dose- and timing-sensitive, because excessive mTORC1 inhibition could interfere with reparative inflammation, cell growth or tissue rebuilding.

#### 5.2.3 2-Hydroxypropyl-β-cyclodextrin (HPβCD)

HPβCD promotes cholesterol mobilization from endolysosomal compartments and has been reported to activate TFEB-dependent lysosome–autophagy transcriptional programs (Song et al., 2014; Yang et al., 2017). By linking cholesterol redistribution with lysosomal adaptation, HPβCD may be particularly relevant to disorders characterized by lipid accumulation or impaired myelin turnover (Lee et al., 2023). However, HPβCD has produced divergent effects on autophagic flux in normal and AD-model mouse brains, indicating that cholesterol mobilization does not consistently translate into improved lysosomal clearance. These context-dependent effects complicate dose selection and extrapolation to traumatic CNS repair.

#### 5.2.4 Adeno-associated virus (AAV)-mediated TFEB/TFE3 gene delivery

A more targeted approach is cell-type-specific delivery of TFEB or TFE3 using engineered AAV vectors. In neurons, TFEB activation enhances aggregate clearance, autophagic flux, and organelle quality control, as shown in Parkinson’s disease and synucleinopathy models (Arotcarena et al., 2019; Decressac et al., 2013). In glial cells, it may improve lysosomal processing of engulfed cargo and modulate inflammatory responses; TFEB–V-ATPase signaling has also been linked to microglial activation in tauopathy (Wang B. et al., 2024). However, sustained TFEB/TFE3 activation may disrupt lipid metabolism, secretion, endocytosis, and immune signaling (Kim et al., 2024). For neural repair, AAV-TFEB/TFE3 is best considered a precision strategy for defined cellular compartments rather than a global lysosome-boosting therapy.

### 5.3 Targeting lysosomal ion channels and membrane repair

#### 5.3.1 Mucolipin synthetic agonist 1 (ML-SA1)

The synthetic agonist ML-SA1 has been shown to rescue Alzheimer-related endosomal–autophagic–lysosomal abnormalities, supporting TRPML1 activation as a strategy to restore late endolysosomal function (Somogyi et al., 2023). ML-SA1 also facilitates α-synuclein aggregate clearance by promoting late autophagy steps, including autophagosome–lysosome fusion and delivery of α-synuclein into acidified vesicles (Pollmanns et al., 2022). Therefore, TRPML1 activation is especially relevant when defective fusion, impaired lysosomal calcium signaling and aggregate retention coexist. In CNS injury, this strategy may be most applicable to stressed neurons or phagocytes with impaired lysosomal fusion but preserved membrane integrity, although its CNS penetration, cell selectivity, and long-term safety remain unclear.

#### 5.3.2 TRPML1–calcineurin–TFEB axis

The molecular basis of the TRPML1–calcineurin–TFEB pathway is described in Section 2.3 (Medina et al., 2015). Therapeutically, it may be useful in cells that need to couple lysosomal stress sensing with expansion of degradative capacity, such as microglia exposed to persistent debris or neurons with chronic proteostatic burden. However, this CNS repair framework still requires direct validation in SCI, TBI, or demyelinating injury models, and the effects of sustained pathway activation may vary with cell type and disease stage.

#### 5.3.3 Lysosomal phosphoinositide signaling

TRPML1 activity is closely related to phosphatidylinositol 3,5-bisphosphate [PI(3,5)P_2_], which is generated by phosphoinositide kinase, FYVE-type zinc finger containing (PIKfyve) (Dong et al., 2010; Vines et al., 2023). PI(3,5)P_2_ can directly regulate TRPML channels and thereby influence the release of lysosomal Ca^2+^, endolysosomal trafficking and membrane dynamic changes (Gan et al., 2022). The regulation of the PIKfyve–PI(3,5)P_2_–TRPML1 axis may affect vesicle fission, endolysosomal transport and terminal cargo degradation (Vines et al., 2023; Wible et al., 2024). However, this pathway needs to be treated carefully, because excessive inhibition of PIKfyve can reduce PI(3,5)P_2_ levels and may lead to late endosome or lysosome swelling and trafficking disorders. Compared with broadly suppressing PIKfyve, precise tuning of this pathway may be more conducive to correcting specific trafficking defects.

#### 5.3.4 Endosomal sorting complexes required for transport (ESCRT), galectin and lysophagy-based membrane repair

Lysosomal membrane damage cannot be solved by acidification alone. By ESCRT-dependent membrane sealing, mild lysosomal membrane injury can be repaired. Thus, this process limits the leakage of lysosomal contents and maintains organelle function (Chauhan and Patro, 2024; Radulovic et al., 2018). More serious or irreparable damage can be recognized by galectins and ubiquitin-dependent machinery, which recruit selective autophagy pathways to remove damaged lysosomes through lysophagy (Gahlot et al., 2024; Maejima et al., 2013). Tectonin beta-propeller repeat-containing protein 1 (TECPR1)-dependent unconventional LC3 lipidation provides an additional layer of membrane quality control (Corkery and Wu, 2024). These mechanisms may be particularly relevant in CNS phagocytes exposed to myelin debris, protein aggregates, and other damage-associated cargo, although direct evidence that pharmacological enhancement of lysosomal membrane repair improves neural recovery remains limited. Selective pharmacological activators and reliable markers of lysosomal membrane repair are also lacking.

### 5.4 Reprogramming lipid turnover and myelin debris clearance

#### 5.4.1 Liver X receptor (LXR) agonists

After demyelination, cholesterol-rich myelin debris must be digested and excess sterols must be exported by macrophages and microglia. Inflammatory phagocyte states may be maintained, and remyelination may be impaired if lipid cannot be effectively removed (Berghoff et al., 2021; Richartz et al., 2025). LXR agonists such as GW3965 and T0901317 target cholesterol-efflux programs including ATP-binding cassette transporter A1 (ABCA1) and APOE, thus linking lysosomal lipid treatment with sterol efflux (Namjoshi et al., 2013; Zhang et al., 2024). In demyelinating conditions, LXR activation is considered to support repair by promoting cholesterol turnover in microglia, astrocytes and oligodendrocytes, while also reducing inflammatory signaling (Richartz et al., 2025). This strategy is especially important when phagocytes accumulate cholesterol esters or lipid droplets after ingesting myelin, because it shifts lipid handling from inflammatory storage toward resolution-associated efflux. However, systemic LXR activation may have a wide range of metabolic effects, so CNS-directed delivery or subtype-selective regulation may be required for translation.

#### 5.4.2 Triggering receptor expressed on myeloid cells 2 (TREM2) agonistic antibodies

TREM2 agonistic antibodies represent an immune-reprogramming strategy that enhances microglial lipid sensing, phagocytosis and myelin debris clearance. In active demyelinating lesions from patients with multiple sclerosis, TREM2 is highly expressed in myelin-laden phagocytes, supporting its relevance to lipid-loaded microglial states (Cignarella et al., 2020). In the cuprizone model, treatment with a TREM2 agonistic antibody promoted myelin debris clearance by microglia and improved remyelination, providing direct experimental support for this strategy in demyelination. Its therapeutic logic is not simply to increase phagocytosis, but to coordinate debris uptake with lysosomal digestion and lipid metabolic adaptation. Recent work on blood–brain barrier (BBB)-shuttled TREM2 agonistic antibodies further supports the feasibility of engaging microglial TREM2 in the CNS, although most translational development has focused on neurodegenerative disease rather than traumatic repair (Schlepckow et al., 2023; van Lengerich et al., 2023). For CNS injury, the key question is whether TREM2 activation can enhance debris clearance without locking microglia into a chronic disease-associated or inflammatory state.

#### 5.4.3 APOE–ABCA1-mediated cholesterol efflux enhancement

The APOE–ABCA1 axis is a key route for exporting cholesterol from lipid-loaded phagocytes. In CNS injury and demyelination-related contexts, LXR activation increases ABCA1 and APOE expression, indicating that this pathway can be pharmacologically engaged to promote cholesterol efflux (Namjoshi et al., 2013; Zhang et al., 2024). The apolipoprotein A-I (ApoA-I) mimetic peptide 5A further supports this concept by enhancing ABCA1-dependent cholesterol efflux, myelin debris degradation, and remyelination (Cantuti-Castelvetri et al., 2018; Vanherle et al., 2022). This approach may be particularly relevant to chronic demyelination and aging, although its safety and efficacy after traumatic SCI remain uncertain.

#### 5.4.4 Interferon regulatory factor 5 (IRF5)-linked regulation of myelin lipid processing

Recent work suggests that IRF5 is involved in myelin clearance and cholesterol metabolism of microglia after demyelination (Montilla et al., 2025). In experimental autoimmune encephalomyelitis (EAE) and lysolecithin-induced demyelination models, the deletion of IRF5 weakened the ability of microglia to process myelin, altered cholesterol homeostasis and was associated with poorer regenerative responses, including damaged oligodendrocyte recruitment into lesions (Montilla et al., 2025). These findings place IRF5 at the interface between inflammatory programming, lysosomal lipid degradation and cholesterol redistribution. The loss of appropriate lipid processing may lead to the persistence of myelin debris, lipid droplet accumulation and the damage of remyelination. Therefore, targeting this pathway may help coordinate the inflammatory state with lipid catabolism during lesion repair. However, since IRF5 is a transcription factor widely involved in the regulation of inflammation, direct inhibition or activation of IRF5 may have context-dependent effects and should not be treated as a simple pro-repair intervention.

### 5.5 Lysosome-responsive nanomedicine and biomaterials

#### 5.5.1 Lysosome-acidifying poly (lactic-co-glycolic acid) (PLGA) nanoparticles

Acidic PLGA nanoparticles can help restore lysosomal acidity. After endolysosomal trafficking, these particles can release acidic degradation products, lower lysosomal pH and improve degradative function in cells with alkalinized lysosomes (Baltazar et al., 2012; Zeng et al., 2019). In models of lysosomal dysfunction, acidic nanoparticles have been shown to restore autophagic flux, enhance lysosomal degradation and reduce aggregate accumulation, supporting their use as tools to directly manipulate the lysosomal chemical environment (Bourdenx et al., 2016; Zhang X. et al., 2023). Compared with soluble small molecules, this strategy offers spatial enrichment within endolysosomal compartments and may be useful for diseases in which lysosomal alkalinization is a central pathogenic event. For CNS repair, cell-type-specific delivery to neurons, microglia or infiltrating macrophages after injury remains unresolved.

#### 5.5.2 Lysosome-targeted liposomes or pH-buffering nanocarriers

Lysosome-targeted liposomes and pH-tuned nanocarriers can be designed to deliver acidifying components, hydrolase-supporting agents or anti-inflammatory cargo directly to endolysosomal compartments. Recent acidic liposomes have been reported to restore lysosomal acidification, improve autophagic flux, and promote protein-cargo degradation (Jeon et al., 2025). More broadly, acid-activated nanoparticles can release acidic components preferentially in dysfunctional lysosomes and restore lysosomal acidification, autophagic function and mitochondrial activity in metabolic disease models (Lo et al., 2024; Zeng et al., 2023). These platforms allow pH correction to be combined with the delivery of small molecules, peptides, or nucleic acids. Their application to CNS repair, however, will depend on lesion-site retention, cell-type selectivity, and long-term safety.

#### 5.5.3 Injury-responsive hydrogels and biomaterial scaffolds for CNS repair

For traumatic CNS injury, lysosome-oriented therapy may also be implemented through injury-responsive hydrogels, nanoparticles or scaffolds that modulate inflammatory phagocytes and local debris clearance. Functional hydrogels have been widely explored in spinal cord injury because they can provide localized and controlled delivery of immunomodulatory agents, cells or trophic factors while reshaping the lesion microenvironment (Ji et al., 2023; Walsh et al., 2022; Zheng et al., 2024). Rather than directly targeting lysosomes, these platforms may indirectly reduce lysosomal burden by limiting oxidative damage, scavenging extracellular damage-associated molecular patterns (DAMPs) and modulating phagocyte activation (Li et al., 2026a; Liu et al., 2026). Representative studies illustrate this distinction. For example, a functional gelatin–hyaluronic acid hydrogel was evaluated primarily through DAMP scavenging, immune-cell recruitment, graft survival and locomotor recovery, whereas an extracellular-vesicle-loaded thermosensitive hydrogel was assessed mainly through microglial polarization, neuronal apoptosis, scar formation and motor recovery (Gao X. et al., 2023; Zhang et al., 2022). The reported mechanistic analyses in these studies therefore focused on inflammatory regulation and tissue repair rather than direct lysosomal readouts, such as lysosomal pH, hydrolase activity, lysophagy or lipid-degradation flux. Accordingly, the lysosome-related effects of these biomaterials should currently be regarded as mechanistically plausible indirect effects rather than experimentally established mechanisms.

### 5.6 Limitations

Lysosome-oriented therapies must be applied with caution because lysosomal enhancement is not always beneficial. Ion homeostasis, membrane stability and hydrolytic enzyme activity may be disturbed by excessive re-acidification; extensive activation of TFEB/TFE3 or inhibition of mTORC1 may affect lipid metabolism, immune response, cell growth and repair programs; and ion-channel regulation or lysosomal membrane repair may have different effects depending on cell type and disease stage (Chauhan and Patro, 2024; Kim et al., 2024; Napolitano et al., 2022). Nanoparticles, viral vectors and biomaterial scaffolds also face challenges such as delivery specificity, long-term safety, dose control and CNS penetration. Many of the approaches discussed above remain preclinical, with limited evidence of *in vivo* target engagement after traumatic CNS injury. Therefore, lysosome-oriented therapy should be guided by functional readouts, including lysosomal pH, enzyme activity, autophagic flux, lipid degradation and membrane repair, rather than lysosomal marker expression alone (Klionsky et al., 2021; Radulovic et al., 2026; Solé-Domènech et al., 2025; Yao et al., 2023).

## 6 Future perspectives and conclusions

Lysosome biology is shifting from descriptive accounts of cargo accumulation to a functional framework. Organelle chemical properties, metabolic recovery, immune regulation and neural repair are linked by this framework. In CNS injury and neurodegeneration, lysosomal dysfunction should be understood as the failure of interconnected modules, including acidification, hydrolase maturation, membrane integrity, ion homeostasis, lipid handling and lysosomal biogenesis, rather than as a single degradative defect (Chou et al., 2025; Nixon and Rubinsztein, 2024).

Future studies need to define lysosomal dysfunction with cell-type and temporal precision. Neurons may mainly fail in axonal autophagic transport and organelle quality control, whereas microglia and macrophages may fail because phagolysosomes are overloaded by myelin debris, oxidized lipids and inflammatory cargo. Therefore, static markers such as LAMP1, LC3-II or p62 should be complemented by lysosomal pH, cathepsin activity, lipid flux, membrane-damage and cargo-degradation assays (Radulovic et al., 2026; Wang B. et al., 2024).

Therapeutically, lysosome-oriented strategies should move beyond global autophagy activation. Broad autophagy induction may increase cargo delivery to already impaired lysosomes, whereas restoring acidification, hydrolase activity, membrane stability, lipid efflux or TFEB/TFE3-mediated lysosomal renewal may more directly recover degradative competence. Nanocarriers, extracellular vesicles and hydrogels may improve local delivery, but excessive lysosomal activation or material accumulation may also cause lysosomal overload and inflammation (Radulovic et al., 2026).

In conclusion, lysosomes should be viewed as active organizers of neural repair rather than passive degradative endpoints. The key question is no longer whether lysosomal dysfunction exists, but which lysosomal module fails, in which cell type, at which disease stage, and whether this change is pathogenic or compensatory. Answering these questions will determine whether lysosome-oriented strategies can become clinically meaningful neural repair therapies (Haney et al., 2024; Zhao et al., 2025).
